# Ketamine Influences CLOCK:BMAL1 Function Leading to Altered Circadian Gene Expression

**DOI:** 10.1371/journal.pone.0023982

**Published:** 2011-08-24

**Authors:** Marina M. Bellet, Marquis P. Vawter, Blynn G. Bunney, William E. Bunney, Paolo Sassone-Corsi

**Affiliations:** 1 Center for Epigenetics and Metabolism, School of Medicine, University of California Irvine, Irvine, California, United States of America; 2 Department of Psychiatry and Human Behavior, School of Medicine, University of California Irvine, Irvine, California, United States of America; Roswell Park Cancer Institute, United States of America

## Abstract

Major mood disorders have been linked to abnormalities in circadian rhythms, leading to disturbances in sleep, mood, temperature, and hormonal levels. We provide evidence that ketamine, a drug with rapid antidepressant effects, influences the function of the circadian molecular machinery. Ketamine modulates CLOCK:BMAL1-mediated transcriptional activation when these regulators are ectopically expressed in NG108-15 neuronal cells. Inhibition occurs in a dose-dependent manner and is attenuated after treatment with the GSK3β antagonist SB21673. We analyzed the effect of ketamine on circadian gene expression and observed a dose-dependent reduction in the amplitude of circadian transcription of the *Bmal1*, *Per2*, and *Cry1* genes. Finally, chromatin-immunoprecipitation analyses revealed that ketamine altered the recruitment of the CLOCK:BMAL1 complex on circadian promoters in a time-dependent manner. Our results reveal a yet unsuspected molecular mode of action of ketamine and thereby may suggest possible pharmacological antidepressant strategies.

## Introduction

Major depressive disorder (MDD) is a serious and recurrent psychiatric illness with a prevalence rate of 6.7% in the United States in any 12 month period [1], [2]. Currently available monoaminergic antidepressant drugs require weeks to months for a full clinical response [3] during which time patients may be at risk for suicidal behavior [4]. Furthermore, a significant number of patients fail to respond to currently available antidepressant medications [5], [6]. There is a growing interest in glutamatergic-based therapeutics as an alternative treatment to conventional antidepressants. Ketamine, a non-competitive high affinity NMDA (N-methyl-D-aspartate) glutamate receptor antagonist, has rapid and robust antidepressant actions in animal models of depression when administered intravenously in low subanaesthetic doses [7].

Clinical studies of low-dose intravenous ketamine report improvement within 24 hours (for review see [8]). An estimated 70% of MDD patients respond to ketamine [9], [10]. Approximately 35% of responders have sustained improvement up to 7 days after a single dose [10], [11] or longer after repeated doses [12], [13].

Accumulating evidence suggests that ketamine alters circadian rhythms, which may be linked to its rapid antidepressant action. Ketamine dampens phase-shifting responses to light [14] and alters diurnal rhythms of the widespread NMDA and AMPA receptors in the SCN. Activation of glutamate receptors increases the mRNA levels of the core clock genes *Per1 a*nd *Per2* [15], [16]. Also, ketamine inhibits the photic induction of *Fos-like* immunoreactivity in the suprachiasmatic nucleus (SCN) (the central regulator of circadian rhythms in mammals) [17]. Both AMPA [18] and NMDA [19] show 24 hour rhythms in the SCN in rodents. Further, the hypnotic efficacy of ketamine increases during the early active phase in mice (11pm) as compared to the early inactive phase (10am) [20]. In humans, ketamine has chronopharmacological properties with maximum anesthetic effects at night [21].

Dysregulation of the circadian system has been linked to depressive disorders. Circadian abnormalities in mood, sleep, temperature, and neuroendocrine function are associated with depression in a subgroup of patients [22], [23]. Diurnal patterns of mood can persist for many months through the course of a depressive episode. Typically, a subgroup of depressed patients will awaken with a severe psychotic depression in the early morning which decreases to an almost euthymic state by evening [24], [25]. Approximately 70–80% of depressed patients report difficulties in sleep ranging from initiating and maintaining sleep to early morning awakening and restlessness [26]. Thermoregulatory deficits associated with depression include elevated nocturnal core body temperature [27], [28]. Disturbances in the hypothalamic-pituitary-adrenal (HPA) axis [29], reflected by alterations in CRH and cortisol levels, are frequently associated with depression [23], [30]. Circadian alterations in depressed patients are variable and can range from phase advances, phase delays, or changes in amplitude. However, consistent abnormalities are the phase advances of physiological events including the shortening of the latency of the first rapid eye movement (REM) stage, early awakening and an early peak of adrenocorticotrophic secretions [23], [27], [30].

Circadian rhythms are generated by a set of core clock genes, which include three period (encoding the proteins PER1, PER2 and PER3), two cryptochrome (encoding CRY and CRY2), two *Bmal* (encoding BMAL1 and BMAL2) and two *Clock* (encoding CLOCK and NPAS2) genes. These critical components of the mammalian clock machinery operate as transcriptional regulators organized in interlocked transcriptional and post-translational feedback loops. A major feedback loop involves CLOCK and BMAL1 which act as a heterodimer to rhythmically drive the transcription of *Per* and C*ry* genes. The PER and CRY proteins accumulate and dimerize in the cytoplasm, then translocate into the nucleus where they bind to CLOCK:BMAL1 to inhibit their own transcription. Importantly, this regulatory system controls also the expression of many clock-controlled genes (CCGs) through promoter elements called E-boxes located in their regulatory regions. Since about 10–15% of all cellular transcripts are controlled by the clock [31], it is obvious that CCGs are involved in a large array of biological functions, including the control of several physiological functions, like aging [32]–[36] and cellular metabolism [37], [38]. Finally, the cyclic control of a large fraction of the genome implicates global changes in chromatin remodeling [31]. A key discovery towards the understanding of how the circadian machinery may participate in chromatin remodeling demonstrated that CLOCK also possesses intrinsic histone acetyltransferase (HAT) activity [39], [40] that regulates circadian expression through direct chromatin remodeling.

A question to be addressed relates to how the activation of intracellular signaling pathways is integrated at the level of the circadian transcriptional machinery. Environmental cues, primarily light, can reset the daily phase of internal molecular rhythms. Light signals are transmitted from the retina via the retinohypothalamic tract (RHT) to the SCN. The RHT neurons release glutamate in the ventrolateral SCN. Activation of the NMDA receptor initiates a cascade of events leading ultimately to the induction of immediate-early genes and clock genes [41], [42]. The potential significance of these complex interactions is that ketamine may act at specific sites within the circadian transcriptional machinery to promote circadian phase shifts, although thus far there is no direct evidence of this.

Recent work has implicated the mammalian target of rapamycin (mTOR) as a potential site for ketamine's rapid antidepressant actions. When mTOR is blocked, ketamine's antidepressant effects in animal models of depression are abolished [43]. Other actions of mTOR include the attenuation of phase-delaying responses to early night-light in the SCN. Blocking mTOR significantly dampens the light-induced expression of the circadian PER1 and PER2 proteins [44], [45].

The aim of this study was to investigate ketamine's effects on circadian gene expression. We demonstrate that ketamine acts by modulating the activation potential of the CLOCK:BMAL1 complex by interfering with their recruitment to chromatin at a target circadian promoter. Our results reveal a yet unsuspected molecular mode of action of ketamine and thereby suggest possible pharmacological antidepressant strategies.

## Results

### Ketamine inhibits CLOCK:BMAL1 transactivation potential

**Figure 1:**
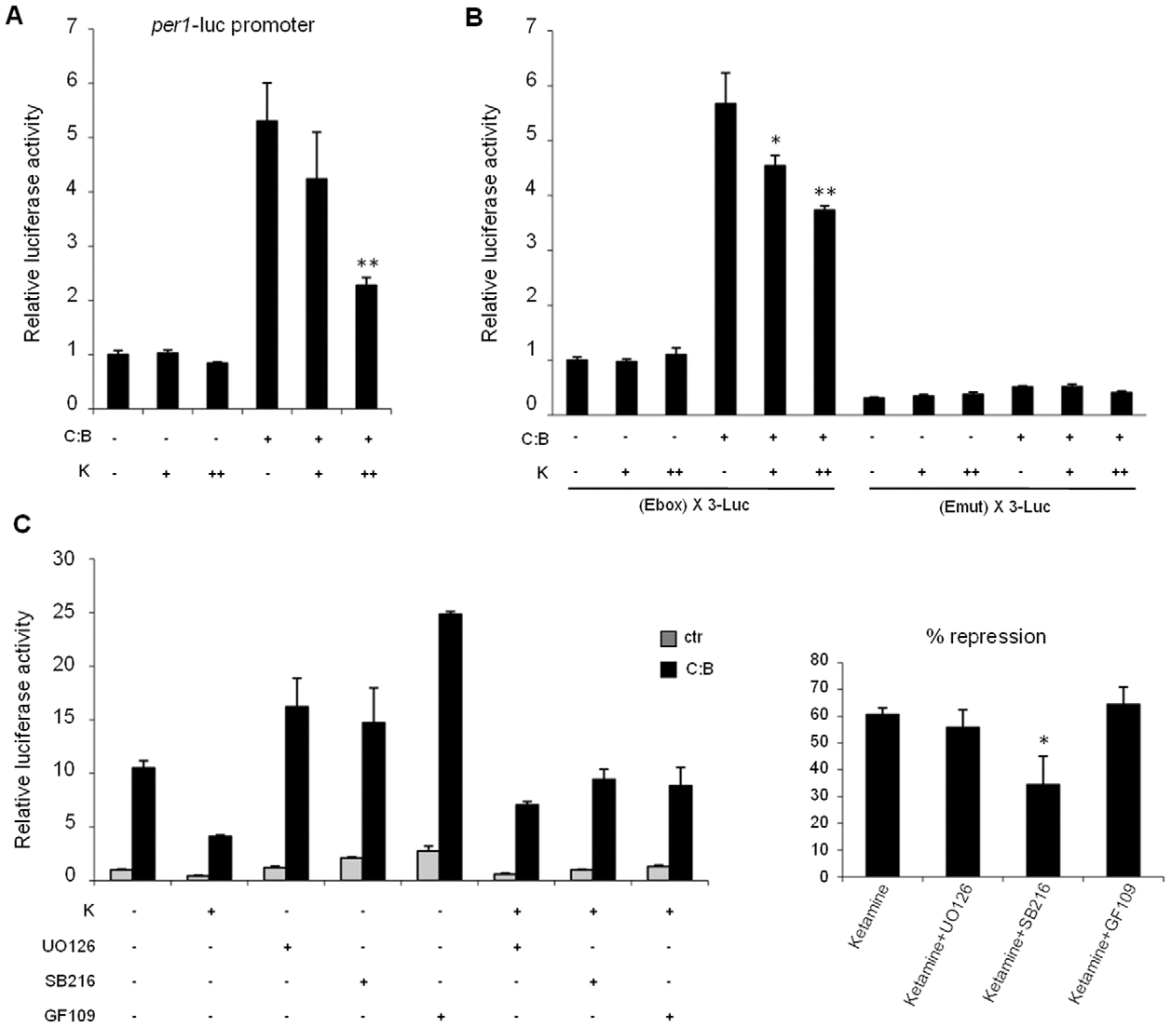
Ketamine treatment represses CLOCK:BMAL1 transactivation potential on the *mPer1* gene promoter. (A) Effect of ketamine on CLOCK:BMAL1 dependent transcription. Vectors expressing CLOCK:BMAL1 (C:B, 25 ng each) were cotransfected with a construct containing the *mPer1* promoter (pGL3-mPer1-Luc, 50 ng) in NG108-15 cells. The total DNA amount was kept constant by adding carrier plasmid DNA. After 6 hs of transfection, cells were treated with increasing amounts of ketamine (K, 18 h, + as 10 mM and ++ as 1 mM). After normalization for transfection efficiency using β-galactosidase activity, reporter gene activities were expressed relative to those of a control transfected only with non-expressing plasmids. All the values are the mean +/− SD (n = 3); (**) p<0.01. (B) The E box promoter element mediates ketamine repression of CLOCK:BMAL1. Experimental conditions were as in A except that reporter constructs containing three copies of the E box consensus sequence (pGL3 promoter (E box) X3 LUC) or its mutated form (pGL3 promoter (Emut) X3 LUC) were used. All values are the mean +/− SD (n = 3); (*) *p*<0.05, (**) *p*<0.01. (C) Ketamine repression of CLOCK:BMAL1 involves activation of GSK3β. Experimental conditions were as in B except that the kinase inhibitors UO126 (ERK kinase inhibitor), SB216763 (GSK3β kinase inhibitor) and GF109203 (PKC kinase inhibitor) were applied to cells together with ketamine treatment (K, ++ as 1 mM). % of repression of CLOCK:BMAL1 transactivation with ketamine alone or ketamine with different kinase inhibitors is shown. All values are the mean +/− SD (n = 3); (*) *p*<0.05.

To explore the effect of ketamine on circadian physiology we decided to monitor how it may influence clock gene expression. To do so, we analyzed whether ketamine could modulate CLOCK:BMAL1-driven transcription. A reporter constituted by the *mPer1* gene promoter fused with the luciferase gene was ectopically expressed in NG108-15 neuronal cells by transient transfection. As previously reported [46], co-expression of CLOCK:BMAL1 results in activation of the *mPer1* promoter. The effect of ketamine was analyzed by treating the transfected cells with increasing doses of ketamine. While ketamine has no effect on the basal *mPer1* promoter activity, it significantly induced a dose-dependent reduction of CLOCK:BMAL1-driven activation (Fig. 1A).

Since the activity of the *mPer1* promoter has been shown to be influenced by signaling pathways which utilize transcription factors other than CLOCK:BMAL1 [42], [47], [48], we sought to investigate whether the effect of ketamine is mediated uniquely by the E-box elements. We transfected a reporter containing three consensus E-box sequences located upstream of the luciferase gene. Importantly, ketamine significantly affected CLOCK:BMAL1-driven transcription by acting on the E boxes. This effect was abolished when the E-boxes were mutated to impair CLOCK:BMAL1 binding (Fig. 1B). Thus, the effect of ketamine is specifically directed on the CLOCK:BMAL1 complex.

The effect of ketamine appears to be dose-dependent. While the doses used in the in vitro experiments are obviously higher than that used in human antidepressant studies, they are comparable to other studies in various neuronal and not neuronal cell types [49]–[53].

To better understand the molecular mechanisms implicated in the effect of ketamine on CLOCK:BMAL1-driven transcription, we treated transfected NG108-15 cells with various protein kinase inhibitors in the presence or absence of ketamine. Interestingly, the ketamine-induced repression of CLOCK:BMAL1 is reduced after treatment with the GSK3β inhibitor, SB216763, while it remains unaltered after treatment with the mitogen-activated protein (MAP) kinase kinase MEK1/2-specific inhibitor, UO126 or the PKC inhibitor, GF109203 (Fig. 1C). This data suggests that the inhibitory effect of ketamine on circadian function involves GSK3β, a kinase that has been already implicated in the regulation of various circadian proteins [54]–[58] and which has been linked to the regulation of depressive disorders [59], [60].

### Ketamine alters circadian gene expression

**Figure 2:**
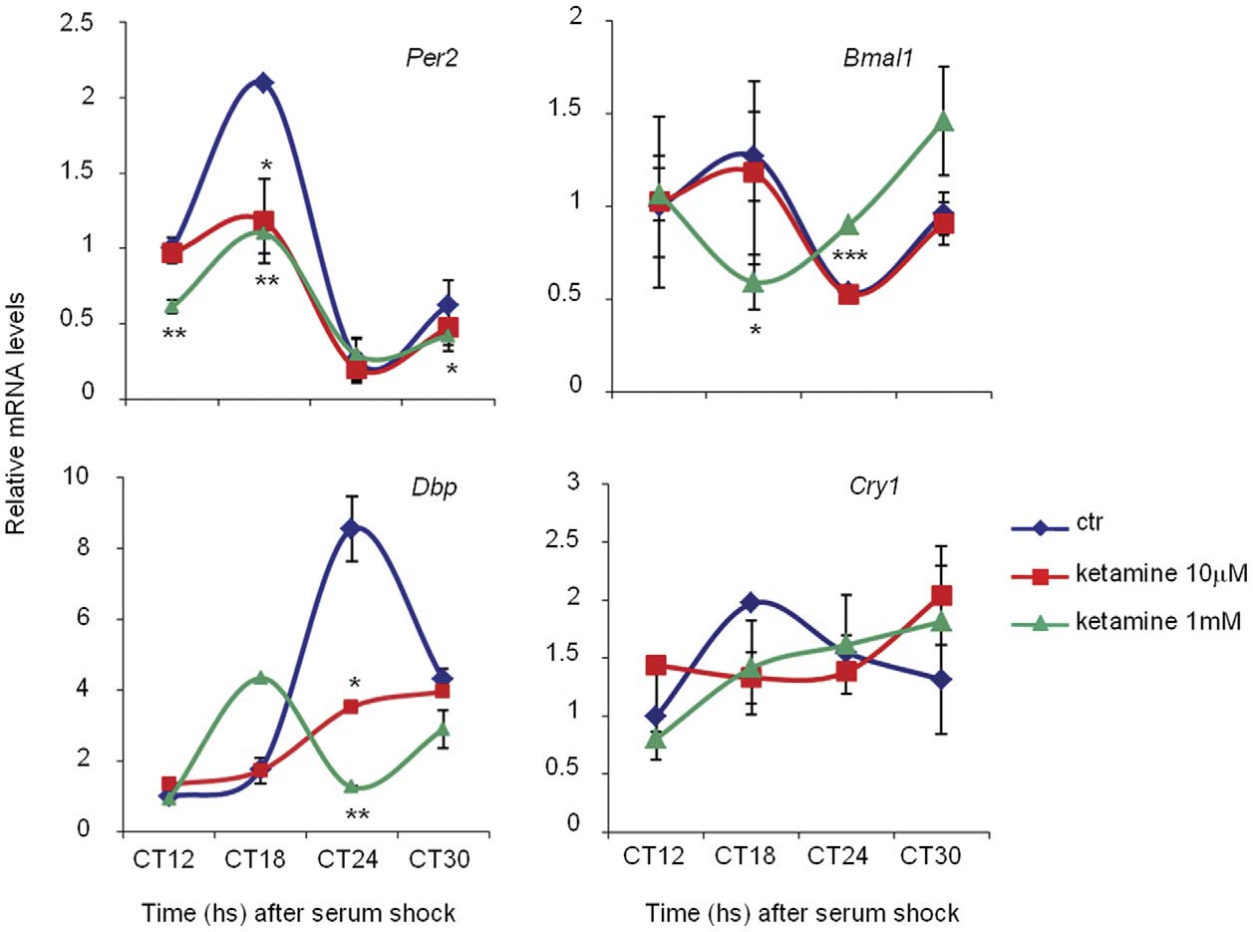
Altered circadian expression of clock genes and CCGs after ketamine treatment. *Per2*, *Dbp*, *Bmal1* and *Cry1* mRNA expression profiles in WT MEFs, untreated or treated for 1 h with ketamine (10 mM and 1 mM) at circadian times CT11–12, CT17–18, CT 23–24, CT29–30 after serum shock, were analyzed by quantitative PCR. The values are relative to those of 18S mRNA levels at each CT (circadian time). Time 12 (CT12) value in untreated cells was set to 1. All values are the mean +/− SD (n = 3); (*) *p*<0.05, (**) *p*<0.01, (***) p<0.001.

The finding that ketamine treatment inhibits the transcriptional potential of CLOCK:BMAL1 prompted us to investigate how this effect might influence circadian gene expression. Circadian transcription can be readily studied in mouse embryonic fibroblasts (MEFs), which are synchronized in response to a 2 hrs exposure to 50% horse serum. MEFs generated from wild-type mice were thereby treated with ketamine at various times after serum-induced synchronization. RNA was extracted from cells arrested at various times after the serum-shock and analyzed using quantitative PCR. The analysis revealed that ketamine alters the expression of clock genes (*Bmal1*, *Per2*, C*ry1*) and of the clock-controlled *Dbp* gene during the circadian cycle. Remarkably, *Per2*, C*ry1* and *Dbp* gene expression showed a dose-dependent reduction of the amplitude of the oscillation while *Bmal1* and *Dbp* expression appeared to be phase-shifted (high dose ketamine) (Fig. 2). Thus, the influence of ketamine on CLOCK:BMAL1 (Fig. 1) is paralleled by a significant effect on the circadian expression of endogenously oscillating genes.

### Chromatin recruitment of CLOCK:BMAL1 to circadian promoters is affected by ketamine

**Figure 3:**
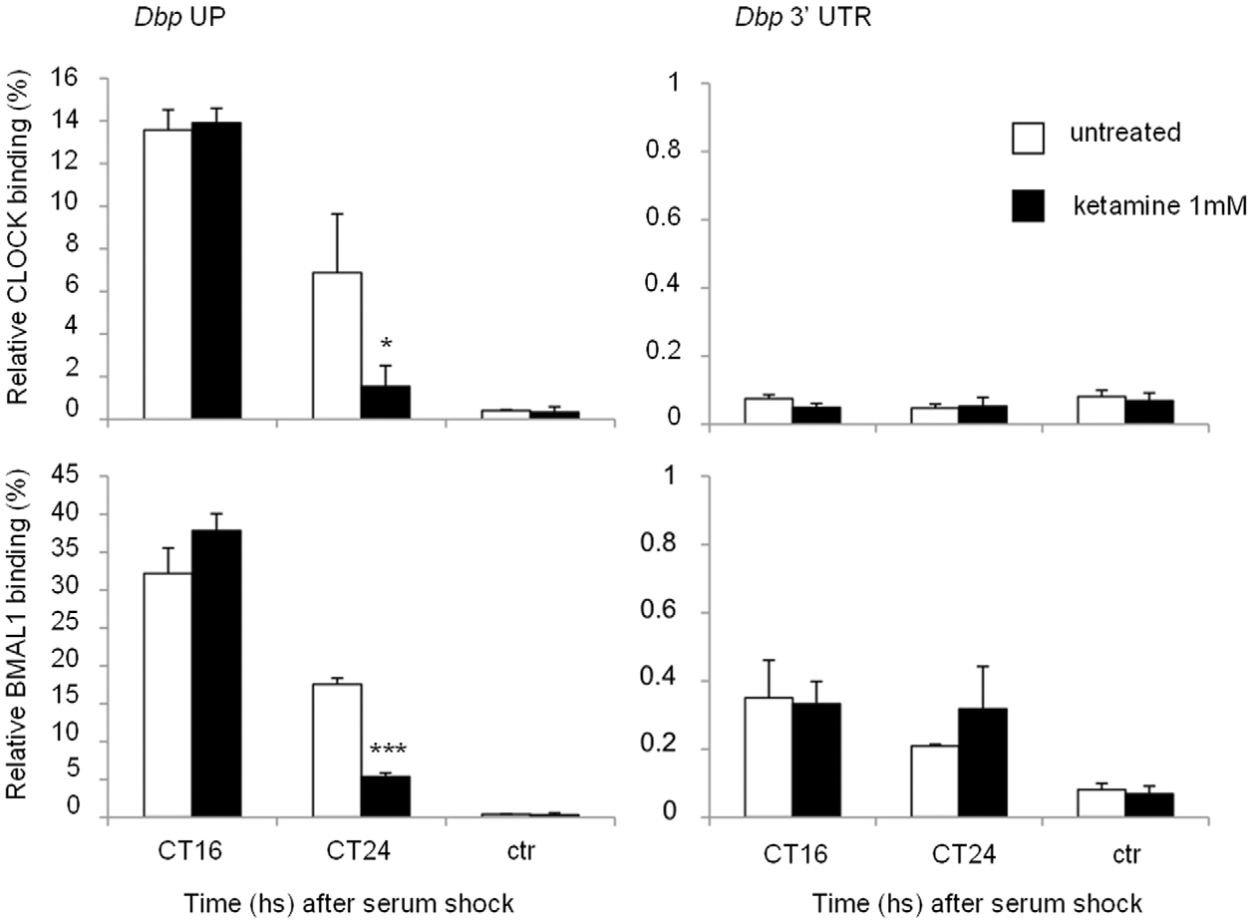
Altered CLOCK and BMAL1 circadian recruitment to CCGs promoter after ketamine treatment. Cross-linked cell extracts were isolated at the indicated time points after serum shock from MEFs, not treated or treated with ketamine (1 mM) for 1 h before each time point. The samples were subjected to ChIP assay with anti-CLOCK, anti-BMAL1 and anti-IgG, and analyzed by quantitative PCR with primers for *Dbp* promoter. Control IgG (ctr) and *Dbp* 3′UTR (3′ region of *Dbp* promoter) primers were used as controls for immunoprecipitation and PCR, respectively. All the values are the mean +/− SD (n = 3); (*) *p*<0.05, (***) *p*<0.001.

Based on the significant effect of ketamine on circadian transcription, we sought to decipher the mechanism by which ketamine exerts its action. As ketamine appears to act directly on CLOCK:BMAL1 (Fig. 1) and does not affect the stability of CLOCK and BMAL1 proteins (not shown), we reasoned that ketamine could influence their circadian recruitment to chromatin. Thus, we tested CLOCK:BMAL1 recruitment by dual cross-linking chromatin immunoprecipitation (ChIP) assays on the regulatory region of the *Dbp* clock-controlled gene (Fig. 3a). ChIPs were performed on MEFs after serum shock synchronization and ketamine treatment, and analyzing the E-box in the *Dbp* UP region, previously validated to bind CLOCK:BMAL1 [61]. As expected, CLOCK and BMAL1 were both recruited in a time-dependent manner to this region. Importantly, ketamine reduced the recruitment of both CLOCK:BMAL1 at CT24, which parallels the decrease in gene expression observed in response to ketamine (Fig. 3A). No recruitment was observed at the 3′ UTR region of the same promoter (Fig. 3B). This result constitutes the first case of a pharmacological treatment that alters the recruitment of the circadian machinery to a target promoter. These results confirm that ketamine operates on circadian gene expression by directly modulating some key components of the circadian machinery.

## Discussion

The data from this study indicate that ketamine alters circadian rhythms through the inhibition of CLOCK:BMAL1 mediated transcription. The effect on transcriptional activation is paralleled by the effect on circadian gene expression in cultured cells: we found time-dependent changes in clock gene expression by analyzing *Per2*, *Cry1*, *Dbp* and *Bmal1*. The effect of ketamine is observed on clock genes with different phases of oscillation. Antiphase oscillations in *Bmal1* and *Dbp* expression have been described previously in MEFs [62]. As can be seen in figure 2, our control data confirmed these findings as both *Bmal1* and *Dbp* were expressed antiphasically. A dampening of *Per2* mRNA oscillation at both ketamine doses was seen at CT18 post serum shock, lasting for a total of 12 hours before returning to normal levels. No phase shifts were seen in *Per2* mRNA levels over the course of 30 hours. In contrast, low and high doses of ketamine appeared to disrupt *Cry1* oscillations to produce a reduction in amplitude. Although statistically not significant, we have observed a trend in which high doses of ketamine produced a gradual rise in *Cry1* mRNA levels, which persisted throughout the cycle. Low doses of ketamine are instead associated with a sharper rise in *Cry1* levels at CT 24, suggesting a possible trend toward phase delay. Importantly, the effects on circadian expression elicited by short-lasting treatments (1 hour) are reminiscent of the consequences of ketamine treatment in vivo.

Additional in vitro and in vivo studies using neuronal cell lines and neuronal tissues from mice treated with ketamine at different times of the circadian cycle will help further our understanding.

We also elucidated the pathways that are primarily involved in ketamine's effect by blocking kinases that play a role in the transmission of intracellular signals. We observed that the specific blocking of the Akt/GSK3β pathway reduced the effect of ketamine on the circadian system. This initial observation is of interest for future investigations in this direction. Furthermore, while treatment with other kinase inhibitors had no apparent effect on ketamine under the experimental conditions used, we cannot exclude that other pathways could be activated during treatment with ketamine [63]. Future *in vivo* experiments will help clarify these mechanisms.

To our knowledge this is the first study to demonstrate ketamine's effects on the core clock genes and could suggest an additional mechanism of action underlying clinical and preclinical observations of ketamine-induced rapid antidepressant actions. Although several actions of ketamine have been described, including effects on synaptic plasticity, AMPARs, NMDARs, and most recently on mTOR, to our knowledge no studies have investigated the possible interaction of ketamine with the circadian system.

Circadian rhythm abnormalities have long been associated with the pathophysiology of depressive illness. Shifting circadian rhythms using non-invasive interventions such as bright light, sleep phase advance, and/or sleep deprivation therapies induce rapid improvement in a subgroup of patients. It is hypothesized that these treatments act by resetting abnormal circadian rhythms [8], [22], [23]. Drug interventions including antidepressants and mood stabilizers may accomplish this by synchronizing clock gene activation. Lithium, for example, lengthens circadian rhythms [64] and is thought to act by inhibiting GSK3β [55], [65]. GSK3β phosphorylates various clock proteins, affecting their stability to modulate the negative feedback loop to CLOCK:BMAL1 [54], [56], [57]. Importantly, we have reported here that the action of ketamine appears to be specifically blocked by a GSK3β inhibitor (Fig. 1). This is consistent with data showing that inhibition of GSK3β is necessary to ketamine's rapid antidepressant action in a mouse model of depression (learned helplessness) [66].

Data from mammals suggest that 2–3 hours are required post-phase shifts to reset behavioral rhythms [67]. This effect was demonstrated in cultured SCN cells using real-time imaging data from transgenic mice carrying a luciferase reporter gene. NMDA applied to the SCN either induced phase delays at peak oscillation times or phase-advances during the trough phase of luminescence demonstrating that the direction and magnitude of the NMDA-induced phase shifts are dependent on the circadian phase [67]. While we have not addressed whether the effect of ketamine on circadian gene expression is mediated by NMDA, it is important to underscore that NMDA receptors are expressed in different types of non-neuronal cells [68]–[72]. Future studies will help to better elucidate the mechanisms of action of ketamine on the circadian system in neuronal and non-neuronal tissues. As Clock mutant mice display manic-like behaviors, which can be reversed by lithium [73], it will of interest to explore how ketamine influences circadian behavior. Our findings favor a scenario in which these rapid changes in circadian clock gene expression may, in part, play a role in the rapid antidepressant actions associated with ketamine.

## Materials and Methods

### Reagents and plasmids

Ketamine-HCl, UO126, SB216763, GF109203 were purchased from Sigma. Plasmids expressing myc-tagged *mClock* and FLAG-myc-tagged *mBmal1* were described previously [74]. Plasmids expressing both β-galactosidase (pGL3-lacZ) for transfection control, and luciferase (luc) for luminometry based expression, pGL3-m*Per1*-Luc promoter, pGL3-Ebox X3-luc, and pGL3-Emut X3-luc, were described previously [47].

### Antibodies

Antibodies against CLOCK and rabbit IgG were from Santa Cruz Biotechnology, antibody against BMAL1 was described earlier [40].

### Cell culture

NG108-15 cells were grown in DMEM (4.5 g/L glucose) supplemented with heat-inactivated 10% fetal bovine serum (FBS) and antibiotics and cultured at 37°C in 5% CO_2_. MEFs were generated from wild type C57BL/J mice and cultured in DMEM (4.5 g/L glucose) supplemented with 10% FBS and antibiotics.

### Transient transfection and luciferase assay

Cells were transfected with BioT (Bioland) according to the manufacturer's protocol. Cell extracts were subjected to a luminometry-based luciferase assay and luciferase activity was normalized by β-galactosidase activity.

### Quantitative RT-PCR

Each quantitative real-time RT-PCR was performer using the Chromo4 real time detection system (BIO-RAD). The PCR primers for murine Per2, Cry1, Dbp, Bmal1 mRNA, 18S rRNA, Dbp UP promoter region, Dbp 3′ UTR promoter region were described previously (61). For a 20 µl PCR, 25 – 50 ng of cDNA template was mixed with the primers to final concentration of 300 nM and 10 µl of iQ SYBR Green Supermix (Bio-Rad), respectively. The reaction was first incubated at 95°C for 3 min, followed by 40 cycles at 95°C for 30 s and 60°C for 1 min.

### Chromatin Immunoprecipitation (ChIP) assays

Dual cross-linking ChIP assay [75] was used. Briefly, after 2 h of serum shock with media containing 50% horse serum, cells were incubated with serum-free medium for the indicated time. Ketamine treatment was added 1 h before the collection. Then, cells were washed three times with room temperature PBS and PBS with 1 mM MgCl_2_ was added. Disuccinimidyl Glutarate (DSG, Pierce) was added to a final concentration of 2 mM for crosslinking and incubated 45 min at room temperature, formaldehyde was added to a final concentration of 1% (v/v) and cells incubated for 15 min for dual crosslinking, and glycine was added to a final concentration of 0.1 M and incubated for 10 min to quench formaldehyde cross-linking. After harvesting, cells were lysed in 500 µL ice-cold cell lysis buffer (50 mM Tris/HCl pH 8.0, 85 mM KCl, 0.5% NP40, 1 mM PMSF, 1x protease inhibitor cocktail (Roche)) for 10 min on ice. Nuclei were precipitated by centrifugation (3000 g for 5 min), resuspended in 600 µL ice-cold RIPA buffer (50 mM Tris/HCl pH 8.0, 150 mM NaCl, 1 mM EDTA pH 8.0, 1% Triton X-100, 0.1% SDS, 0.1% sodium deoxycolate, 1 mM PMSF, 1x protease inhibitor cocktail) and incubated on ice for 30 min. Sonication was performed to obtain DNA fragments 100–600 bp in length.
